# Unraveling chronic kidney disease in children: a surprising manifestation of celiac disease

**DOI:** 10.3389/fped.2024.1384591

**Published:** 2024-04-24

**Authors:** Iuliana Magdalena Starcea, Ingrith Miron, Ancuta Lupu, Ileana Ioniuc, Mirabela Alecsa, Alice Azoicai, Ionela Daniela Morariu, Valentin Munteanu, Vasile Valeriu Lupu, Adriana Mocanu

**Affiliations:** ^1^ Mother and Child Medicine Department, “Grigore T. Popa” University of Medicine and Pharmacy, Iasi, Romania; ^2^ Faculty of Pharmacy, “Grigore T. Popa” University of Medicine and Pharmacy, Iasi, Romania; ^3^ Faculty of Medical Bioengineering, “Grigore T. Popa” University of Medicine and Pharmacy, Iasi, Romania

**Keywords:** celiac disease, chronic kidney disease, children, IgA nephropathy, diabetic nephropathy

## Abstract

Celiac disease, firstly described in children, is a type of T-cell enteropathy that occurs in individuals genetically predisposed to gluten exposure. The estimated global prevalence of celiac disease is continuously increasing. Although, traditionally, celiac disease was diagnosed in children with failure to thrive and digestive issues, it is now recognized that may present with a wide range of symptoms beyond gastrointestinal ones. Celiac disease continues to pose significant challenges due to the continuous advancement of knowledge in understanding its pathophysiology, diagnosing the condition, managing its effects, and exploring potential therapeutic approaches. The prevalence of celiac disease is increased among individuals with chronic kidney disease, also. The most frequent associations are with diabetic nephropathy, IgA nephropathy and urolithiasis. A gut-kidney axis has been recognized to play a significant role in chronic kidney diseases. This literature review aims to review the chronic renal pathology associated with celiac disease, with emphasis on childhood.

## 1 Introduction

Celiac disease is an autoimmune condition that affects the entire body, causing disruptions in the internal balance and resulting in specific changes in the tissue structure of the small intestine. These changes include significant villi damage with increased crypt formation. Clinically, the disease presents a diverse range of symptoms, spanning from gastrointestinal issues like diarrhea, bloating, weight loss, and abdominal discomfort to non-digestive symptoms such as iron deficiency anemia, delayed puberty, and oral ulcers. These symptoms collectively stem from varying levels of malabsorption (1, 2).

Celiac disease is correlated with a number of autoimmune disorders, including type 1 diabetes and thyroid disease, which are classified as “associated conditions” or conditions that exhibit an elevated incidence but are not directly attributable to gluten consumption (2).

CD is a type of T-cell enteropathy characterized by an abnormal immune response to gluten, a protein found in wheat, barley, and rye. The estimated global prevalence of CD ranges from 0.7%–1.4% (3). CD can affect individuals of all ages and may present with a wide range of symptoms beyond just gastrointestinal problems (4).

CD continues to pose significant challenges due to the continuous advancement of knowledge in understanding its pathophysiology, diagnosing the condition, managing its effects, and exploring potential therapeutic approaches. As awareness of CD has increased, healthcare professionals have become better at identifying atypical and asymptomatic cases. Thus, more adults are now being diagnosed with CD, indicating that the disease's clinical spectrum is broader than previously understood (5).

The identification of tissue transglutaminase as an immunological target has offered a reliable first-line diagnostic test for CD (6). The introduction of screening for CD has enabled the diagnosis of certain cases even in the absence of typical digestive symptoms (7). According to recent studies (8, 9), the occurrence of CD is twice as common in children compared to adults, and 1.5 times higher to women than men. Although small intestinal biopsy remains the “gold standard” in the diagnosis of CD, current serological tests, such as tissue transglutaminase, endomysial gliadin and de-amidated peptide antibodies, have become more important in the diagnosis of the disease (5). The long-term effects of using this new no-biopsy approach on adherence to a gluten-free diet have yet to be evaluated (1, 10).

Currently, the only treatment for CD remains the strict gluten-free diet throughout life, which leads to improved quality of life, relief of symptoms and prevention of reducible complications, such as intestinal lymphoma and intestinal adenocarcinoma (11, 12). CD also presents a variety of extraintestinal manifestations, which affect the skin, liver or joints, nervous system or kidney (13, 14).

The prevalence of CD is increased among individuals with chronic kidney disease (CKD), as evidenced by current studies (15). Also, an increased risk of end-stage renal disease (ESRD) has been associated with CD (16). The most frequent associations are with diabetic nephropathy, IgA nephropathy and reno-urinary lithiasis (17, 18).

A gut-kidney axis has been recognized to play a significant role in chronic kidney diseases. Disruption of the gut barrier can lead to the abnormal entry of bacterial lipo-polysaccharide (LPS) endotoxin into the circulation. This abnormal entry contributes to uremic toxicity and systemic inflammation, which are factors implicated in the development and progression of chronic kidney diseases (19, 10).

This literature review aims to review the chronic renal involvement associated with celiac disease, with emphasis on childhood.

## 2 Material and methods

The scientific literature was browsed using Scopus, PubMed, and Embase da-ta-bases by searching the following keywords: “celiac disease,” “chronic kidney disease,” “T-cell enteropathy,” and “children,” in various combinations. The search was limited to open-access articles published in English within the last 10 years. Studies that mostly focused on creating a full definition of celiac disease in children were included. These studies were especially interested in the extradigestive symptoms of the disease, with a focus on kidney involvement. Articles that were vague and did not focus on specific aspects of the subject were excluded from the review process, but they were still used for general informational purposes.

## 3 Diabetic kidney disease and celiac disease in children

Diabetic kidney disease (DKD) has been observed concurrently with the increased incidence of diabetes mellitus (DM) in children. The kidney disease associated with DM in children and adolescents is represented by persistent albuminuria, arterial hypertension, the progressive decrease in the glomerular filtration rate, with the possibility of evolution towards the final stage (20). The association of type 1 diabetes (T1DM) with CD is known, and its prevalence in children varies from 3%–12% (20). A significant study was developed in Sweden, where patients with T1DM are routinely screened for CD. The risk of kidney disease in patients with type 1 diabetes mellitus and coexisting CD was evaluated in a cohort of 954 patients with both T1DM and CD, comparing the prevalence of CKD with that of patients diagnosed with T1DM alone (21). The authors found similar percentages of patients who developed chronic kidney disease in both groups.

Previous studies discussing the incidence of CKD in patients with T1DM and CD are limited to the adults and report conflicting results. Gopee et al., showed that the gluten-free regimen in children with CD + T1DM has a renoprotective effect, by decreasing albuminuria, compared to those who only have T1DM (22). The same HLA predisposition to CD influences the development and progression of albuminuria (23).

The Genetics of Kidneys in Diabetes (GoKinD) study included 1,879 individuals with long-term evolution of T1DM, half of them with diabetic nephropathy. Involvement of DRB1 is consistent with the known immunological processes in the pathogenesis of diabetic nephropathy, especially in the Late Autoimmune Diabetes of Adults, less in children. There is an inverse and separate relationship between the risk of CD and the age at which diabetes begins, with a greater risk observed in children under 4 years old compared to those over 9 years old. The mechanism of association of these two diseases involves a shared genetic background: HLA genotype DR3-DQ2 and DR4-DQ8 (23, 24).

Another study investigating the risk of ESRD in CD patient showed that the effect of type 1 diabetes on the risk was only marginal (16). The review by Boonpheng et al. (18) demonstrated that CD can be independently related to kidney dysfunction, regardless of the development of type 1 diabetes.

CD is also an independent risk factor for microvascular complications related to diabetes, but also for macrovascular complications (25) especially an increased risk the intima-medial thickness of the carotid arteries (26). The established association between CD and microvascular complications in diabetes is another independent argument in favor of screening for CD in the patient with T1DM (27). However, the mechanism behind how CD increases the risk of microvascular disease in patients with T1DM remains unclear and is likely to involve multiple factors (28). One such factor is the association of unrecognized CD with elevated homocysteine levels, which may be attributed to deficiencies in folic acid and other B vitamins, contributing to endothelial dysfunction (29). A recent study indicated that supplementing vitamin B in individuals with CD was linked to significantly lower homocysteine levels, potentially reducing the risk of vascular disease (30).

## 4 IgA nephropathy and celiac disease

IgA nephropathy (IgAN), identified almost 60 years ago by Berger (31), is one of the most common glomerulonephritis reported worldwide (32). IgA antibodies are produced by B cells in the mucosal tissues, such as the lining of the gut and airways. When these antibodies are activated, they can form immune complexes that can deposit in the kidneys and damage the glomeruli (33). Immunoglobulin A, the hallmark of mesangial deposits of IgAN, is the main immunoglobulin in mucous secretions (34). Recent data suggest that the dysregulated gut mucosal immunity, genetic conditioning, gut dysbiosis, and diet play a combined role in development and progression of IgAN (35). People who have a genetic predisposition to IgA nephropathy in high-risk groups may be able to be prophylactically screened for the CD and closely monitored by immunohistochemical methods or identified by genetic testing. The prognosis for IgA nephropathy varies depending on the severity of the disease. However, with early diagnosis and treatment, most people with IgA nephropathy can live a normal life (36). The link between a dysregulated gut-associated lymphoid tissue (GALT) and IgAN was hypothesized following the observation of a higher association between IgA nephropathy and celiac disease (37). However, in people with IgAN, GALT can become dysregulated, leading to the production of abnormal IgA antibodies. These antibodies can then deposit in the kidneys, causing inflammation and damage. The link between GALT and IgAN has been supported by a number of studies. A genome-wide association study (GWAS) found that most loci associated with the risk of IgAN are also associated with immune-mediated inflammatory bowel diseases, maintenance of the intestinal barrier and regulation of response to gut pathogens. This suggests that exposure to intestinal microbes may play a role in the development of IgAN, particularly in people who are genetically predisposed to the disease (38).

The production of mucosal IgA is induced both by mechanisms dependent on T lymphocytes and by mechanisms independent of them (39). After priming, B cells migrate to the effector zone of the mucosa-associated lymphoid tissue (MALT), located in the lamina propria of the mucosa, where they release dimeric IgA, composed of two IgA molecules and a connecting chain (35). Secretory IgA has enhanced bacteriostatic effects due to antimicrobial peptides, such as defensins, which are secreted into the intestinal lumen.

In the last decade the role of gut microbiota in patients with IgAN has been intensive investigated (40). Toll-like receptors (TLRs), such as LPS (TLR4) and lipoteichoic acid (TLR2), are significantly expressed on mucosal epithelial cells and recognize the molecular patterns of microbes (41). Gut microbiota signals the gut injury response through TLRs (42). An altered intestinal barrier facilitates increased absorption and circulation of LPS (43). Studies have indicated the presence of increased intestinal permeability in patients with IgAN (35, 40).

The causal association between IgAN and CD was analyzed in prospective study, by screening patients with IgAN for CD and explored the utility of analysis of IgA anti-TG2 antibody deposits. IgA anti tTG2 Ab colocalization study in kidney biopsies of patients with suspected celiac associated IgA nephropathy were found, but a small proportion of patients with IgAN have associated CD (44).

The gluten-free diet has been shown to be effective in treating IgA nephropathy in people with celiac disease. The gluten-free diet helps to reduce the production of IgA antibodies, which can lead to the disappearance of IgA deposits in the glomeruli and improvement of the condition of the glomeruli (45).

Children with IgA Vasculitis or just IgAN have increased expression of TRL4 mRNA in the periphery lymphomononuclear cells, which was correlated with signs of activation of innate immunity and proteinuria (35, 46). These observations lead to the hypothesis that increased intestinal permeability in intestinal microbes it triggers, by activating TLR4, immune system activation detected in IgAN. This mechanism correlates with systemic inflammation and oxidative stress (35).

In celiac disease, increased intestinal permeability is a distinctive feature (47). Many studies suggest the existence of analogies between intestinal barrier disruption in patients with CD and those with IgAN. Gluten aggravates IgAN through the interaction of gliadin with CD89, favoring the formation of the IgA1–sCD89 complex (35, 45). In both IgAN and celiac disease, the transferrin receptor (TFR) is associated with transglutaminase 2 (TG2), both of which are present in enterocytes and mesangial cells.

**Figure 1:**
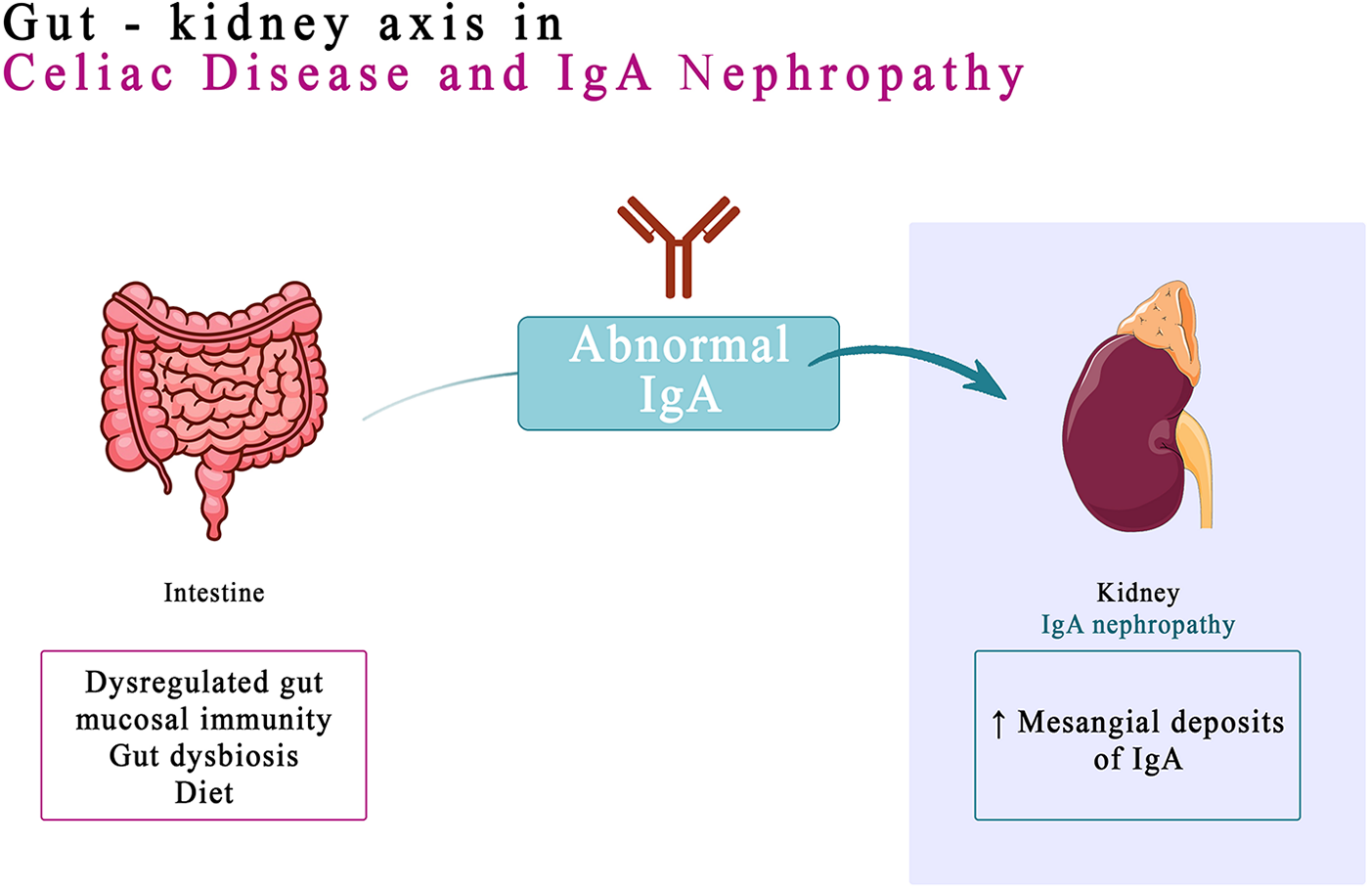
Pathogeny of IgA nephropathy in celiac disease.

Tight junctions represent the foremost junctional complex located at the apex of cellular structures, serving to establish a polarized arrangement that segregates the apical and basolateral poles of the cells (48). In celiac disease, increased intestinal permeability results from disruption of intestinal epithelial cell tight junctions (TJ), leading to increased absorption of dietary antigens (48, 49). Gliadin, the lectin component of gluten, is deamidated by TG2 and presented to T cells in the context of HLA-DQ2 or HLA-DQ8 molecules on dendritic cells or macro-phages. Interaction with CD4+ T cells produces a TH1 response, generating proinflammatory cytokines, resulting in mucosal atrophy and a further increase in intestinal permeability. This process also generates a TH2 response, with the production of antibodies by B lymphocytes in the GALT (the lymphoid tissue associated with the intestinal mucosa). Thus, the production of IgA and IgG against gliadin and TG2 is stimulated. Most patients with CD associate the HLA-DQ2/DQ8 haplotype and show anti-gliadin and anti-TG2 antibodies (35) (Figure 1).

In patients with IgAN, it is hypothesized that a similar intestinal permeability mechanism might be active, with the release of anti-gliadin IgA into the circulation to form macromolecular aggregates with IgA1/sCD89, which ultimately deposit in the mesangial tissue (35). The detection of IgA anti-TG2 deposits in the mesangial tissue is a reliable technique for establishing an association with CD in these patients (43). The correlation is also supported by studies that show the remission of IgAN associated with CD in patients subjected to a gluten free diet (GFD) for at least 5 years (49). The link between IgA nephropathy and CD should be kept in mind. CD may be associated with a progressive evolution of CKD towards the end stage (50).

## 5 Urolithiasis and celiac disease

The first report of the association between CD and urolithiasis in children was made by Ogilvie et al. in the 1970s. The authors found that over half of CD patients had hyperoxaluria (51). Recent studies show increased risks of recurrent lithiasis, especially oxalic, in patients with CD (52, 53).

Urine supersaturation with calcium, phosphates, oxalates or cystine is necessary but not sufficient to explain the formation of calculations. The absorption defect of one or more compounds in the gut, or a defect in the renal handling of these compounds, causes supersaturation, primum movens in initiating the formation of kidney stones. Practically, the imbalance between the factors promoting and inhibiting the formation of stones (malabsorption, increased absorption of oxalates, cystine, calcium, vs. decreased absorption of citrate, magnesium, pyrophosphate at the intestinal level) leads to urinary supersaturation with the possibility of initiating crystallization on a support of cellular detritus. Alternatively, it can be speculated that autoantibodies present in CD may accompany urolithiasis. Relevant for this hypothesis, was a study in which anti-SS-A anti-bodies were detected in patients with urolithiasis, who developed Sjögren's syndrome. In another study, the antinuclear antibodies were positive in patients with hypocitraturia known favoring factor for urolithiasis (53–55).

Disturbances in the gut microbiome and metabolome may thus be determinants of early-onset disease and may explain the association between antibiotics and nephrolithiasis. In addition, decreased butyrate production and decreased oxalate degradation among people with early-onset kidney stones has been proven in studies. Roseburia species, (1% of all bacteria present in the gut microbiome) produce butyrate, a short-chain fatty acid that is a mediator of inflammation, helping to maintain the gut mucosal barrier and regulating the expression of oxalate transporters in the gut (56). Dysbiosis contributes to the loss of this species of beneficial bacteria, leading to the mediation of local inflammation, with increased oxalate absorption, which explains hyperoxalic lithiasis (57).

## 6 Other nephropathies and celiac disease

A few studies have reported the occurrence of other glomerulopathies (except IgAN) in the context of celiac disease, including membranous nephropathy and membranoproliferative nephropathy (4). Interestingly, in four case reports, membranoproliferative nephropathy showed improvement following a gluten-free diet (18).

The coexistence of CD and nephrotic syndrome is an exceptionally uncommon occurrence (58). In celiac disease, exposure to gliadin triggers the release of zonulin from enterocytes. High serum levels of zonulin activates protease-activated receptor 2 (PAR2) in a paracrine manner, and determine the disruption of the actin cyto-skeleton and cell-cell junctions in the intestinal epithelium (59, 60). PAR2 is also expressed on podocytes. The increased levels of serum zonulin induced by gluten ingestion may result in enhanced ligand binding to the PAR2 receptor on podocytes. Consequently, alterations in the podocyte cytoskeleton can impact cell motility and attachment to the glomerular basement membrane, potentially leading to proteinuria (61). Focal segmental glomerulosclerosis (FSGS) is a cause of nephrotic syndrome in children and adolescents, as well as an important cause of renal failure in adults (62). Isolated cases of membranous nephropathy (MN) associated with CD have been documented. Giménez et al. reported a series of five children with nephrotic syndrome who developed CD. In the renal biopsy performed revealed lesions of the MCD type or mesangial glomerulonephritis with IgM deposits, without IgA deposits, whose coexistence with CD is exceptional (63). The connection between CD and MN is associated with the common autoimmune pathogenesis, proven in studies by the remission of proteinuria simultaneously with the disappearance of IgA anti-tissue transglutaminase antibodies, sometimes without immunosuppression, only with antiproteinuric therapy and a gluten-free regime (64, 65).

Mukta Mantan et al. cited a 2007 study from the Mario Negri Institute for Pharmacological Research in Milan, Italy, which examined the relationship between bread consumption and the risk of kidney cancer. Individuals with the highest bread consumption had a 94% greater likelihood of developing kidney cancer than those with the lowest bread consumption (66).

## 7 Distal renal tubular acidosis and celiac disease

Renal distal tubular acidosis (RTA) is a significant cause of refractory rickets and stunting in children. In rare cases, distal RTA may be autoimmune in nature, suggested by its association with other autoimmune conditions such as Sjogren's syndrome, systemic lupus erythematosus (SLE), and Hashimoto's thyroiditis (67).

The hereditary form of distal renal tubular acidosis (RTA) is primarily caused by autosomal recessive mutations in genes that encode subunits of the vacuolar H + ATPase. These mutations lead to impaired transporter function in the renal tubules (67, 68). The acquired form of distal RTA is more frequently observed in adults and is often associated with autoimmune conditions, notably Sjögren's syndrome (69, 70).

Although the association of CD with Sjögren's syndrome and distal RTA was previously reported in adults (71), the association of distal tubular acidosis with celiac disease, without other autoimmune determinations, was reported only in a study in children (67).

## 8 Urinary tract infection and celiac disease

Increased urinary tract infection (UTI) incidence in patients with CD has been attributed to an associated disorder of the urinary system, including tract motility impairment, urinary bladder dysfunction, changes in intestinal bacterial flora that predispose to urinary tract contamination, reduced immune defenses against infections, or altered immunity (72). At the same time, the reverse relationship can also be valid. A simple urinary tract infection can acutely trigger an immune reaction that causes a celiac crisis in a predisposed individual (73). Celiac crisis is the hyperacute manifestation of CD, sometimes the onset of a previously undiagnosed gluten enteropathy.

## 9 Final considerations

**Figure 2:**
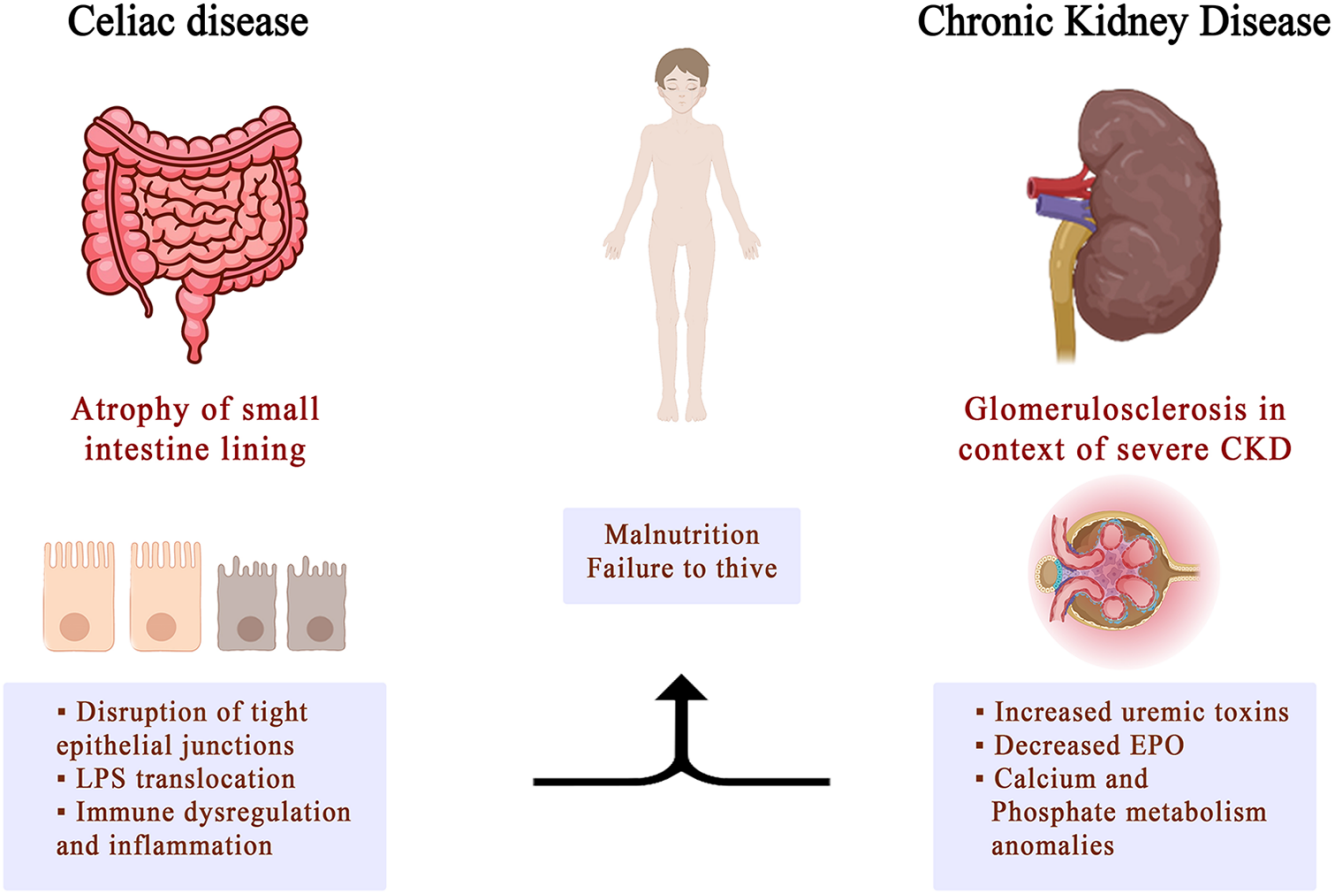
Implication of celiac disease in chronic kidney disease.

CD is often accompanied by various extraintestinal manifestations, which classifies it as a systemic disease rather than a gastrointestinal condition. Many of the extraintestinal manifestations of CD involve the kidneys. These include conditions like urolithiasis and crystal-induced kidney disease, membranoproliferative glomerulonephritis, IgA nephropathy, or an increased risk for progressing to end-stage renal disease (Figure 2).

However, there is limited knowledge about the risk of kidney disease in children with celiac disease. To date, only a few studies have investigated the risk of renal disease among patients with CD, especially adult patients, and there are no current recommendations for screening for renal involvement in these patients. More studies and pro-longed follow-up are needed to evaluate the connections between celiac chronic kidney diseases and celiac disease, especially in childhood where the experience in limited.
